# Influence of Citric Acid on the Bond Strength of Beech Wood

**DOI:** 10.3390/polym13162801

**Published:** 2021-08-20

**Authors:** Goran Mihulja, Vjekoslav Živković, Dominik Poljak, Bogoslav Šefc, Tomislav Sedlar

**Affiliations:** 1 Faculty of Forestry and Wood Technology, University of Zagreb, 10000 Zagreb, Croatia; gmihulja@sumfak.unizg.hr (G.M.); vzivkovic@sumfak.unizg.hr (V.Ž.); bsefc@sumfak.unizg.hr (B.Š.); 2 Drvodjelac d.o.o., 42240 Ivanec, Croatia; poljak@drvodjelac.hr

**Keywords:** chemical modification, citric acid, beech wood, bond strength, PVAC glue

## Abstract

In this study, beech wood (*Fagus silvatica* L.) has been chemically modified with citric acid (*Acidum citricum*) and sodium hypophosphate (SHP) as the catalyst and gradually thermo-condensed in the dryer. Afterwards, wetting angle, surface energy, and shear strength of glued joints of modified and unmodified wood were determined. Testing of the bond strength according to standard EN 204 and comparison between modified and unmodified samples were executed. The adhesive used for bonding samples was polyvinyl acetate (PVAC), commonly used for gluing solid wood panels. Testing material was divided into three groups (dry, wet, and wet conditioned samples), within which statistical analysis was performed, and the significance of the differences between the modified and unmodified samples was determined. Surface energy is correlated with the bond strength, indicating that modification with citric acid negatively affects the adhesive properties of beech wood. A reduction in the bond strength of modified wood glued with PVAC glue compared to unmodified wood was determined. All the results indicate that the modified samples do not meet the minimum requirements for EN 204 bonded with PVAC glue. Therefore, it will be necessary to conduct further studies using other types of adhesives to investigate whether modified wood might be suitable for gluing.

## 1. Introduction

Chemical protection can be executed as a chemical modification of wood based on the reaction of hydroxyl groups of basic wood components (cellulose, hemicellulose, and lignin) with ethers, acetates, esters, etc. [1,2,3,4]. One of the processes of chemical modification is acetylation. Research has shown that acetylation of wood can significantly improve many properties, such as dimensional stability (70–80%), natural durability up to eight times against brown rot, its compression strength of about 10%, and enhances its natural resistance to UV light/radiation [2,5,6,7,8].

Another type of modification that was extensively investigated was using dimethyloldihydroxyethyleneurea (DMDHEU). This type of modification results in increased dimensional stability and durability [8,9,10,11,12,13,14,15,16] and reduced mechanical and sorption properties of modified wood. Mamiński et al. [17] reported: “that the treatment resulted in a significant improvement in the properties of the material: 45% density gain, water absorption, and thickness swelling reduced by 48 and 43%, respectively, a 2.3-fold increase in hardness as well as 3.8-fold and 3.6-fold increase in bending strength and modulus of elasticity, respectively”. The mechanical properties of modified wood depend on several factors: wood species, temperature and duration of modification, the concentration of chemicals, and type of catalyst [7,11].

Recently, environmentally friendly non-formaldehyde agents are increasingly used. Polycarboxylic acids, which include citric acid-CA and 1,2,3-4 butane tetracarboxylic acid-BTCA, have been used to improve the dimensional stability of wood panels because they are formaldehyde-free [18,19]. The citric acid (*Acidum citricum*) is widespread and can be found in various plants’ fruits, roots, and leaves. For example, lemon juice contains 7% citric acid. Citric acid is also found in milk, blood, and urine, so it is also a physiologically essential acid. Due to inexpensive industrial production and the environmental justification of Polycarboxylic acids (PCA), increased research on wood modification is being undertaken with these acids [20,21,22,23,24]. By applying citric acid (CA), the first anhydride disappears between two adjacent carboxyl groups. The anhydride formed reacts with the hydroxyl groups of the cellulose, and a second anhydride is formed between the carboxyl groups. This additional possibility of binding anhydrides with hydroxyl groups causes crosslinking and good bond stability and thus processing stability. The optimal crosslinking temperature of citric acid with cellulose is 180 °C. Katović et al. [25] esterified fir and beech with citric acid, and the results showed a good improvement in the dimensional stability of wood. Ando and Umemura [26] concluded that citric acid bonds with wood components and that the bonds improve the wood molding properties, although polysaccharides are damaged slightly in this process. Šefc [11] impregnated fir and beech wood samples with citric acid and enhanced dimensional stability; compressive strength parallel to the grain was not affected but reduced its micro tensile strength.

Modification of wood with CA did not improve the weathering resistance of modified wood, since CA modifies cellulose but not lignin [27]. Weathering degradation often occurs on the lignin polymer photochemically by exposure to UV light. As a result, the cellulose fibers are loosened and can be easily washed off from the wood surface, which subsequently leads to a rough surface of weathered wood [28].

Due to its complex structure, gluing wood is quite different from gluing other materials. Blomquist [29] demonstrated that even under controlled conditions, the gluing process does not guarantee that the maximum strength of the joint will be obtained. In addition to wetting and adhesion, the type of adhesive also plays a significant role in gluing [30].

Strength and durability are the primary mechanical properties of a glued joint. Therefore, it is essential to distinguish the bond strength from the joint bond strength [31]. The strength of a bonded joint is the strength of the structure that makes up that joint, and it is influenced mainly by forces that can lead to separation [32]. In the wood industry, it is widely believed that the glued joint must be stronger than the material to be glued. In this case, reliable determination of the fracture fraction through wood is of great importance in defining gluing quality [33,34,35]. Unfortunately, wood breakage is a widespread occurrence with quality glued joints. Although an extensive fraction of wood fracture is generally desirable, when it occurs, the measured strength is lower than the actual joint strength [36].

Citric acid has been used as a primary bonding component for a wide range of wood composites such as particleboard, plywood, wood-based molding, fiberboard, and veneer-based panels [28,37,38]. Citric acid-bonded particleboard showed inferior physical properties (water absorption and thickness swelling) and mechanical properties (internal bond strength, modulus of rupture, and modulus of elasticity) compared to particleboard bonded with formaldehyde-based resin [39]. The performance of CA bonded particleboard can be enhanced with increasing citric acid content. The authors also reported that the fungal and termite resistance of citric acid-bonded particleboard is improved.

The aging of wood causes significant changes in the dimensions of wood in products. Therefore, the application of innovative technologies and methods that reduce dimensional changes is of great importance for the use of wood. However, any kind of modification aimed at improving properties such as dimensional stability resulted in some negative effects [1,5,15,27]. Since a glued joint is used in almost all wood products, it is justified to investigate the impact of wood modification with citric acid on the adhesive properties of the surface and to know the real potential of its use.

As one of the most commercially important wood species in Europe, beech wood is used as a reference wood species by European standards. Furthermore, its good technological properties can be applied for various wood industry processes (veneer, chairs, tables, wood panels, etc.) [8,27]. Previous research [7,15] demonstrated an improvement of biological durability of beech wood modified with CA. These results suggest that beech wood modified with CA could be used in places with increased humidity and outdoors. For example, it could even substitute fir and spruce wood in construction where smaller dimensions are required.

This paper aims to determine the wetting properties of beech wood surfaces modified with citric acid, its surface energy, and its suitability to form adequate and durable glued wood joints.

Changes in wetting properties and surface energy of modified wood will be comparable to changes in mechanical properties of PVAC bonded joints. It is expected that there will be a significant strength loss within modified samples as it was noticed in contact with wood coatings [27].

## 2. Materials and Methods

### 2.1. Material Preparation

The test panels used for this research were made from solid beech wood without any visual defects. Average wood density was 730 kg/m^3^, average ring width 2.8 mm, and moisture content 10%. Beech wood panel samples with dimensions 170 × 110 × 5 mm were made for gluing strength investigation (EN 204 [40], EN205 [41]) and small samples of 25 × 20 × 7 mm to investigate wood surface energy. All samples were conditioned in a laboratory for seven days before any further treatment (modification, gluing, sawing, or testing).

Half of the panels were impregnated with 7% aqueous citric acid with the addition of 6.5% sodium dihydrogen hypophosphite (SHP) as a catalyst. The impregnation process was performed according to Šefc et al. [12]. The impregnation cycle consisted of treating the samples in an initial vacuum of 2 kPa in dry conditions for one hour. The vacuum chamber was then filled with citric acid solution and kept under the constant pressure of 200 kPa for 17 h. After that, vacuuming in solution was applied again at a pressure of 2 kPa for three hours, followed by reapplying a pressure of 200 kPa for two hours. The final treatment was carried out in a vacuum of 2 kPa without a solution for 0.5 h.

**Figure 1:**
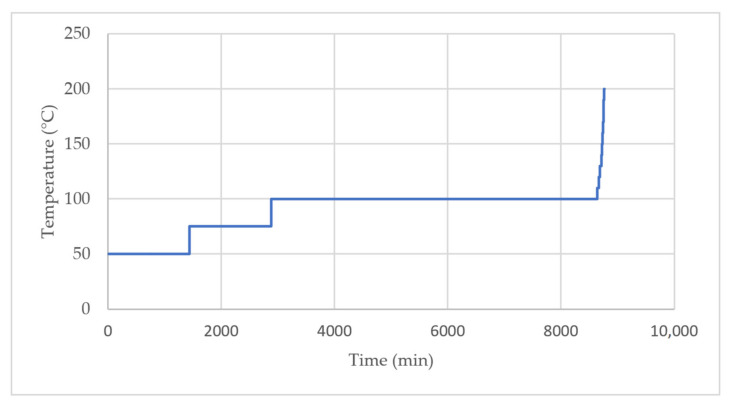
Temperature input timeline for thermal condensation.

After impregnation, the modified panels were gradually thermally condensed in an oven to ensure the action of the appropriate temperature throughout the cross-section in the regime shown in Figure 1. This procedure was applied according to Miklečić and Jirouš-Rajković [27]. Untreated samples served as a reference.

Before preparing testing samples, all the panels were sanded with sandpaper 120 in the fiber direction, with specific pressure below 2 N/mm^2^. Sanding was performed equally in both directions parallel to the fibers until the surface was refreshed entirely with the planning traces (cycloids) removed. This way, the surface was activated, and at the same time, the possibility of uneven thickness of the adhesive layer in the joint was reduced.

### 2.2. Wetting Angle and Surface Energy

The surface energy calculation was based on wetting angles. To determine the surface energy of wood, three liquids with their surface tension values shown in Table 1 were used.

**Figure 2:**
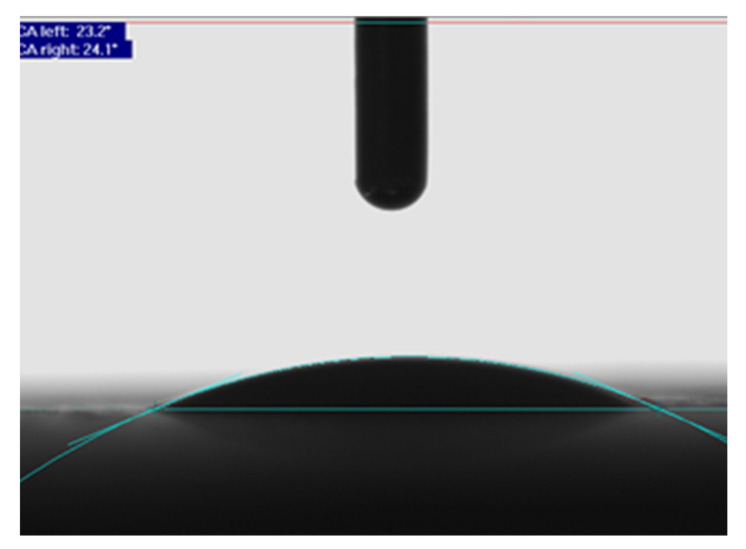
Illustration of measurement of the contact angle.

**Figure 3:**
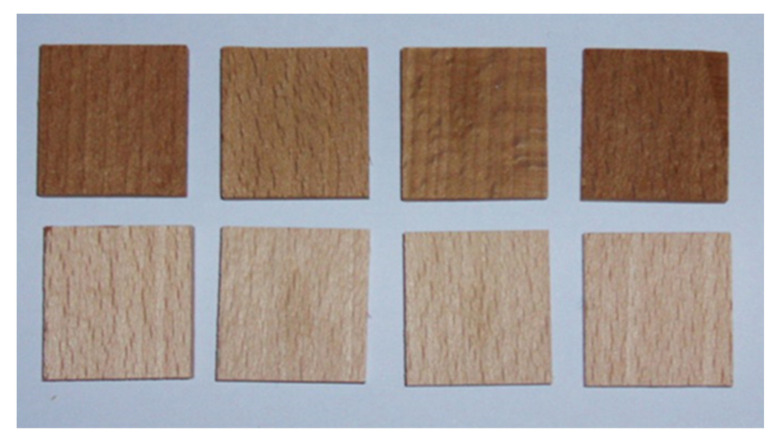
Samples of modified (**first row**) and unmodified beech wood (**second row**).

The wetting angle was measured by the “Sessile drop” method, which determines the wetting angle by a drop of liquid of known surface tension (EN 828 [42]) (Figure 2). Measurements were performed on five modified and five unmodified small wood samples (Figure 3) with a goniometer “DataPhysics OCA20”.

Liquids were selected according to references from the literature [43]. Drops of measuring fluids of water, formamide, and diiodomethane of volume 2 μL were applied to the wood surface. All measurements were performed within 24 h after machining.

**Figure 4:**
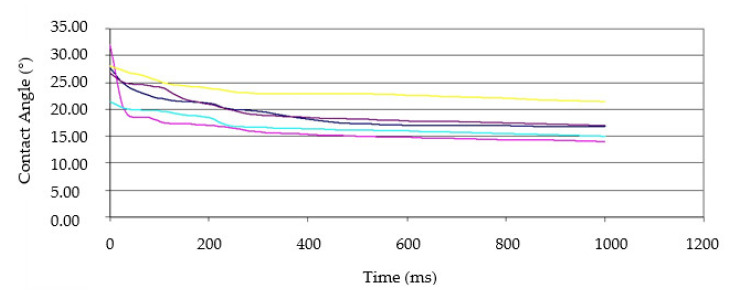
Example of the results of diiodomethane contact angle changes on the modified surface of five samples.

The DataPhysics OCA20 goniometer recorded films of application of drops to the surface and its changes over time (Figure 4). The contact angle of the deposited declines in time was read from the recorded film. Measurements were made at the transition from early to late wood so that the wetting of the surface of early and late wood was equal. Wood samples were selected to have annual rings at an angle of 45 ° in order to obtain a lower variability of wetting angle and avoid uneven spillage of droplets due to the irregular wood structure.

Only one drop of measuring liquid was applied to a specific measuring spot. Particular care was taken to ensure that the drops were applied at the turn of the year and that the surfaces of the drops did not overlap, even if the liquid had evaporated.

As measuring liquid never reaches its stable state on wood, the measurement interval from droplet application to wetting angle stage is obtained by calculating the mean values of the transition points of the contact angle change. In the first phase, the contact angle changes rapidly, while in the second phase, it slows down. The first contact angle of the second phase was used as the wetting angle. An example of contact angle changes is shown in Figure 4.

That means that the wetting angle was determined differently for each measuring liquid according to the time shift from its placement on the wood (Table 1).

The surface energy was obtained according to two measurement principles. The first one was Owens–Wendt–Rabel and Kaelble (OWRK) [44], and the second was Wu’s [45] principle. Both principles divide surface energy into two components: polar and dispersed parts of surface energy. The difference between principles is that Wu applies mathematical instead of geometric data. As a result, it often gives more accurate results than the OWRK method and is more often used to determine higher surface energies.

### 2.3. Bond Strength (EN 204)

Polyvinyl acetate adhesive (Multibond EZ-1) was used for gluing shear test samples. The amount of applied adhesive for gluing the two tiles together was 200 g/m^2^. This application was selected based on experience and coincided with the manufacturer’s instructions, which recommend adhesive application in the amount of 100–210 g/m^2^. The samples were loaded in the press with a constant force of 19,500 N, equivalent to 1 N/mm^2^. Although the manufacturer recommends a 2–3 h pressure time, eliminating internal stresses samples were pressed for 24 h. After adhesion, all the samples were conditioned for seven days in climatic conditions 23 °C and 50%.

**Figure 5:**
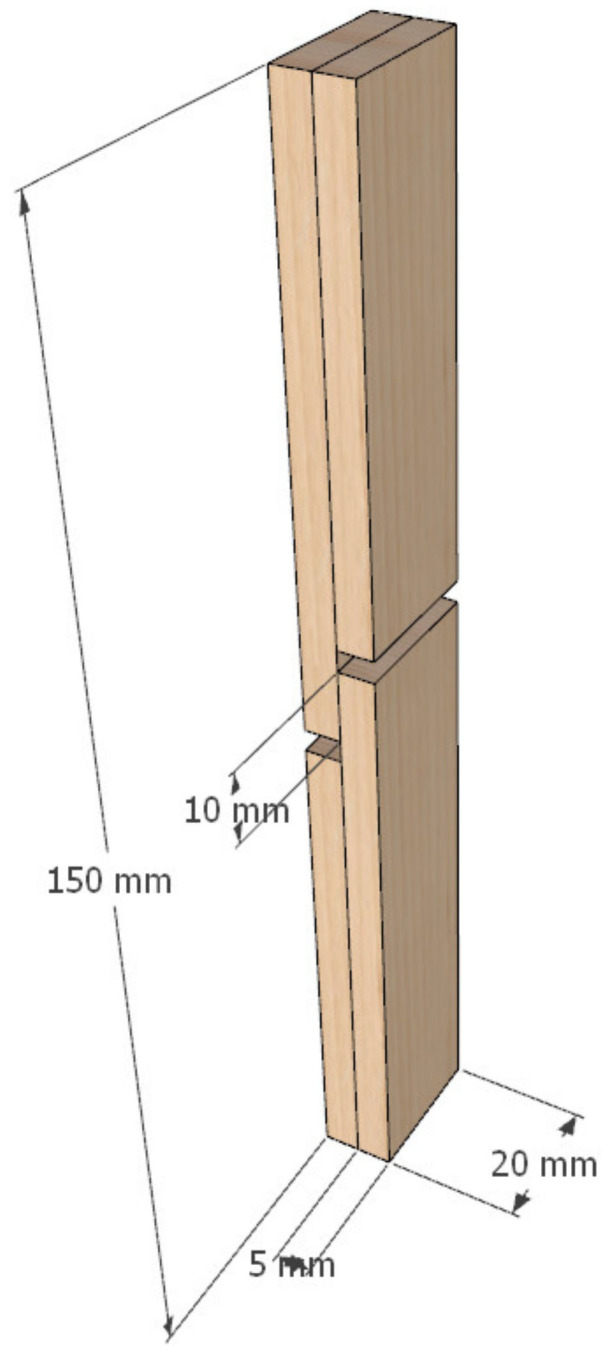
Shear test sample according to EN 205 [41].

A total of 30 glued panels (170 × 110 × 10 mm) were produced. Material for half of the panels (15) was modified before gluing, and the other half was glued as unmodified wood and used as reference samples. Glued panels were then sawn in smaller shear test samples according to EN 205 [41] (Figure 5). Thus, a total of 120 shear test samples were made for testing bond strength (Table 2).

The shear test was performed using a 100 kN universal mechanical testing machine according to EN 205 [41]. The obtained data were processed by Microsoft Excel and Statistica 7.1 for Windows platforms and compared to EN 204 [40] for class D3. This class was used due to the regular use of glued wood products for interiors with frequent short exposure to running or condensing water and/or extended exposure to high humidity and outdoor spaces protected from weather conditions with appropriate structural protection.

If the normality of distribution and homogeneity of variance was satisfied, the differences between the shear strength of the glued joint for individual groups were tested by Leven’s test of homogeneity of variance. If the homogeneity condition was not met (F test and Leven test), the Mann–Whitney U test (2 types/forms of probes) was used.

Graphic representations were created using boxplot graphs. If the group was tested by parametric tests, the arithmetic means and standard deviation were used, and in the case of nonparametric tests, the median of 25th and 75th percentiles were used.

## 3. Results

### 3.1. Wetting Angle and Surface Energy

Wetting angles of unmodified and modified samples were recorded using beech wood and test liquid with known surface tension. By plotting the graphs from five test measurements, it was possible to obtain the measurement interval of the wetting angle for a specific type of test liquid (Table 2).

**Figure 6:**
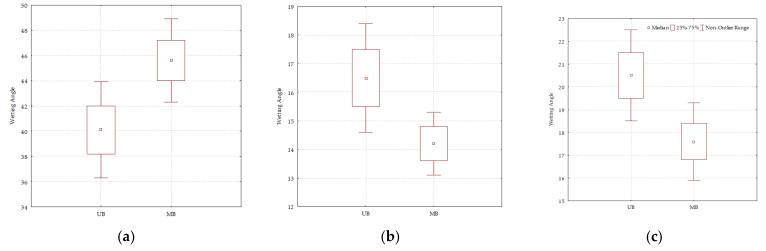
Statistical analyses of measured wetting angles; (**a**) water; (**b**) formamide; (**c**) diiodomethane. UB–unmodified beech wood, MB–modified beech wood.

Table 3 and Figure 6 demonstrate that the wetting angles of water on modified and unmodified beech are statistically significantly different (*p* ˂ 0.05). The wetting angles of formamide and diiodomethane modified and unmodified beech are also statistically significantly different (*p* ˂ 0.05). It can be noticed that in these two measurements, there was a decrease in the wetting angle, which goes in favor of better wetting on the modified wood, and vice versa to the result of water wetting angle on beech. It can be assumed that the modification achieved a higher affinity of wood for a type of liquids with a higher dispersion component.

The surface energy calculation shown in Table 4 was performed according to OWRK and Wu’s measurement principles.

A comparison of the amount of surface energy of citric acid modified beech wood, and unmodified beech shows a higher surface energy of unmodified wood. However, the wetting angles of less polar liquids showed that the wetting is better, not worse. The result of the surface energy calculation indicates that this 15% reduction in surface energy will negatively affect the adhesive properties of beech wood modified with citric acid.

Due to the wood’s biological connection with water and the fact that PVAC adhesive is a water-based system, it can be expected that the result of bonding strength will follow the negative result of wetting measurements with water or reduction of total surface energy. Conversely, adhesives of other chemical affinities could achieve sufficient bonding strength of wood modified with citric acid.

### 3.2. Bond Strength

The presented results are classed into three groups for a more superficial comparison of the obtained results. The groups are arranged so that each modified wood sample has its unmodified reference, depending on the treatment before the gluing strength test.

#### 3.2.1. Group 1—(Unmodified Dry (UD) and Modified Dry (MD))

Modified dry specimens have a statistically significantly lower bond strength compared to unmodified samples (Table 5). In addition, within Group 1, the condition of normality of distribution and homogeneity of variance was not met.

**Figure 7:**
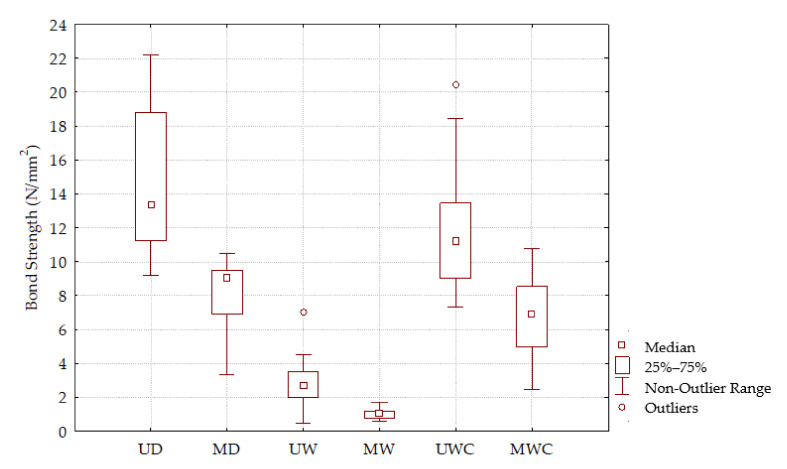
Bond strength of modified wood samples.

In the Group 1 Box plot (Figure 7), the values of the bond strengths of the modified specimens are significantly lower than the strengths of the unmodified samples. When the values are compared with the requirements of EN 204 [40], it can be seen that the bond strength of the modified specimens does not meet the minimum of 10 N/mm^2^ for D1.

The decrease in strength is due to chemical changes on the wood surface modified with citric acid, which is in line with the results of obtained surface energy and the results of the previous research of Miklečić and Jirouš-Rajković [27].

Even though the results of the shear test of modified samples do not meet the requirements of EN 204 [40], greater homogeneity of the adhesive strength results in modified samples (standard deviation of the adhesive strength) was obtained.

#### 3.2.2. Group 2—(Unmodified Wet (UW) and Modified Wet (MW))

Statistically processed data from Group 2 also showed that the condition of normality of distribution and homogeneity of variance was not met (Table 5) and that modified samples based on the Mann–Whitney U test had a statistically significantly lower bond strength compared to the unmodified samples.

In the Boxplot of Group 2 (Figure 7), the difference between the bond strength of unmodified and modified specimens is visible. It is noticeable that the values of adhesive strengths of unmodified samples are significantly higher than the strengths of modified ones which, according to EN 204 [40], do not meet the minimum prescribed requirements.

Although the modified samples of this group do not meet the prescribed standards, the descriptive statistics of Group 2 show greater homogeneity of adhesive strength results in modified samples than in unmodified samples (Table 5—standard deviation of adhesive strength).

#### 3.2.3. Group 3—(Unmodified Wet Conditioned (UWC) and Modified Wet Conditioned (MWC))

Within Group 3, the normality of distribution and homogeneity of variance was met, so the data were tested with a T-test. Statistical analysis shows that the modified samples had a statistically significantly lower strength of the glued joint (Table 5).

In the Boxplot of Group 3 (Figure 7), the difference between the bond strength of unmodified and modified specimens is visible. It is noticeable that the values of adhesive strengths of unmodified samples are significantly higher than the strengths of modified ones which, according to EN 204 [40], do not meet the minimum prescribed requirements.

The descriptive statistics of Group 2 again show greater homogeneity of the adhesive strength results in modified samples than in unmodified samples (Table 5—standard deviation of adhesive strength).

## 4. Conclusions

The difference between the wetting angles of the samples of unmodified beech and beech modified with citric acid presented weaker wetting of the modified wood. This result mainly refers to polar liquids, while the dispersive liquids recorded the opposite effect.

The surface energy of CA modified beech wood with SHP as a catalyst is 15% lower than the surface energy of unmodified samples.

Modification of wood with citric acid (CA) and sodium hypophosphite (SHP) as the catalyst results in reduced bond strength if PVAC glue is applied.

Unmodified dry samples meet all the requirements set by EN 204 [40] for the D3 class of thermoplastic adhesives. In contrast, glued modified wood cannot achieve minimum bond strengths according to EN 204 [40] even for the D1 class requirements.

Citric acid-modified wood is potential material for constructions affected by increased humidity and outdoor use because of its improved biological durability. However, it should be used with caution due to lower surface energies that directly reduce adhesive properties and bond strength, as well as surface adhesion of protective and decorative coatings.

Further research with other types of adhesives is necessary to determine the suitability of citric acid treatment for gluing.

## Figures and Tables

**Table 1:** Surface tension (SFT) of measuring liquids in mN/m, its components (LW, acid, and base), and time frame for contact angle measurement.

Liquid	SFT (total)	SFT (LW ^1^)	SFT (Acid)	SFT (Base)	Modified Samples (sec.)	Control Samples (sec.)
Water [Ström et al.]	72.80	21.80	25.50	25.50	4.50	5.00
Formamide [Van Oss et al.]	58.00	39.00	2.28	39.60	0.45	1.00
Diiodomethane [Erbil]	50.08	50.08	0.00	0.00	0.50	0.50

^1^ LW—equivalent to Lifshitz–van der Waals dispersion component.

**Table 2:** Investigation plan through testing samples.

Semple Type	Sample Mark	Number of Test Samples
Reference samples tested in dry condition (23 °C and 50%)	UD	20
Modified samples tested in dry condition (23 °C and 50%)	MD	20
Reference samples tested after immersed in water for 72 h	UW	20
Modified samples tested after immersed in water for 72 h	MW	20
Reference samples tested conditioned (23 °C and 50%) for seven days after soaking in water	UWC	20
Modified samples tested conditioned (23 °C and 50%) for seven days after soaking in water	MWC	20

**Table 3:** Variance analysis of wetting angles measurements.

Wetting Angle	Analysis of Variance-Marked Effects Are Significant at *p* < 0.05							
	SS Effect	df Effect	MS Effect	SS Error	df Error	MS Error	F	*p*
Water	232.902	1	232.902	1309.630	29	45.159	5.157	0.0303
Formamide	38.002	1	38.002	250.081	28	8.931	4.254	0.0485
Diiodomethane	63.637	1	63.637	397.094	28	14.181	4.487	0.0431

**Table 4:** The surface energy (mN/m) of unmodified and modified beech wood.

Method.	Work			WU		
	Surface energy Total (mN/m)	Dispersive	Polar	Surface Energy Total (mN/m)	Dispersive	Polar
Unmodified wood	72.38	47.62	24.76	77.74	47.66	30.08
Modified wood	62.16	45.07	17.09	65.65	43.58	22.07
Difference (%)	14.12			15.55		

**Table 5:** Average bond strength (BS), standard deviation (SD), coefficient of variation (C.V.), and difference to modified samples.

Specimen Group	Mark	EN 204 Requirements (N/mm^2^)	BS (N/mm^2^)	SD (N/mm^2^)	C.V. (%)	BS Decrease (%)
Group 1	UD	≥10	14.92	4.269	28.61	
	MD		8.12	2.040	25.12	45.58
Group 2	UW	≥2	2.89	1.709	59.19	
	MW		1.06	0.302	28.52	63.32
Group 3	UWC	≥8	11.70	3.701	32.46	
	MWC		6.77	2.565	37.87	42.14

## Data Availability

The data presented in this study are available upon request from the corresponding author.
